# Composition of Flavonoids and Nutritional Evaluation in Leaves of Different Sea‐Buckthorn Germplasm Resources

**DOI:** 10.1002/fsn3.70013

**Published:** 2025-04-01

**Authors:** Yue Yuan, Wentao Yao, Yuqi Wu, Rui Wang, Zeyuan Yu, Junwei Huo, Xingguo Li, Ke Tang

**Affiliations:** ^1^ School of Surveying and Planning Shangqiu Normal University Shangqiu China; ^2^ College of Art and Design Shangqiu Normal University Shangqiu China; ^3^ College of Horticulture Northeast Agricultural University Harbin China; ^4^ Rural Revitalization Research Institute Heilongjiang Academy of Agricultural Sciences Harbin China

**Keywords:** evaluation, flavonoids, germplasm resources, leaf, nutritional components, sea buckthorn

## Abstract

Sea‐buckthorn leaves contain various active components, and research, development, and utilization of sea‐buckthorn leaves have broad application prospects in the fields of food, medicine, and feed. The comprehensive quality of different sea‐buckthorn germplasm resources was evaluated by determining 17 major nutrients, such as flavone composition, polysaccharides, total flavonoids, total polyphenols, and so on in 26 different varieties of sea buckthorn leaves, and then the comprehensive quality of different sea‐buckthorn germplasm resources was evaluated by factor analysis, principal component analysis, and cluster analysis. The results showed that flavonols, mainly quercetin‐like flavonols and isorhamnetin‐like flavonols, were detected in the leaves of 26 sea‐buckthorns. Through comprehensive analysis of its nutritional quality, it was found that the leaves of sea‐buckthorn germplasm resources had highly significant genetic differences and germplasm diversity, and the total polyphenol content, polysaccharide content, VC content, total flavonoids content, and crude fat content of sea‐buckthorn leaves were comparable to those of some commercially available high‐quality famous teas. The higher crude protein content in the leaves of ‘S1’, the higher crude fiber content in the leaves of ‘S4’ and ‘S13’. The higher crude fat content in the leaves of ‘S8’ and the relatively higher polysaccharide content in the leaves of ‘S4’, ‘S22’, ‘S5’, ‘S18’ and ‘S7’ suggest to a certain extent that the leaves of these sea‐buckthorn lines seem to serve as a botanical resource for the extraction of sea‐buckthorn crude protein, crude fiber, crude fat, and polysaccharide, respectively. The contents of flavonoids in ‘S21’ and ‘S24’ leaves were relatively high, and the contents of total polyphenols in ‘S1’, ‘S8’ and ‘S21’ were relatively high, suggesting that these sea buckthorns could be used as plant resources for extracting flavonoids and total polyphenols from sea buckthorns to a certain extent. Factor analysis and principal component analysis showed that ‘S3’, ‘S16’, and ‘S10’ leaves had the highest comprehensive ranking. The results of this study laid a good foundation for the screening of sea buckthorn germplasm resources and the breeding, processing, and utilization of new varieties of sea‐buckthorn leaves.

## Graphical abstract

This study found that the main flavonols detected in the leaves of 26 sea buckthorn varieties were quercetin‐like flavonols and isorhamnetin‐like flavonols. The sea‐buckthorn leaves were categorized into two groups based on their Mg content, and their comprehensive quality was evaluated accordingly. The results showed that varieties ‘S3’, ‘S16’, and ‘S10’ performed the best in terms of comprehensive quality, making them suitable for tea processing.

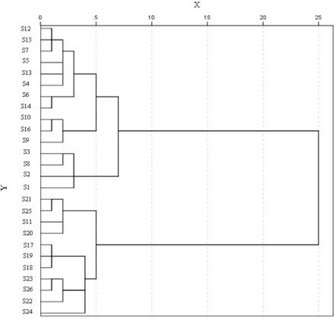

## 1 Introduction

Sea‐buckthorn ( *Hippophae rhamnoides* L.), an ancient miraculous plant, is of great interest because of its tenacity, richness in nutritional active substances, and biological activity (Janceva et al. 2022). Sea buckthorn contains many nutritional active components, such as vitamins, carotenoids, polyphenols, fatty acids, and phytosterols (Gâtlan and Gutt 2021). Moreover, sea buckthorn has many health benefits, such as antioxidant, anticancer, anti‐hyperlipidemic, anti‐obesity, anti‐inflammatory, antimicrobial, antiviral, dermatological, neuroprotective, and hepatoprotective activities (Marsiñach and Cuenca 2019). Its leaves contain a large amount of amino acids, minerals, folic acid, catechins, and other nutrients (Dong et al. 2023). These components exert a wide range of health benefits by exerting antioxidant, anticancer, anti‐inflammatory, antimicrobial, and antiviral effects, as well as exerting protective cardiovascular, dermatological, neuroprotective, and hepatoprotective effects (Ma et al. 2022). Sea‐buckthorn tea, sea‐buckthorn feed, sea‐buckthorn herbal medicine, and sea‐buckthorn health care products prepared from sea‐buckthorn leaves are becoming more and more familiar to more and more people. Sea buckthorn can be processed into high‐quality medicines, food additives, beverages, cosmetics, high‐quality feed and many other products, and its deep‐processed products also have a broad application prospect (Ma et al. 2019).

From the current research status, the evaluation of sea buckthorn leaf resources by previous researchers was only from a single function or trait, while the comprehensive evaluation of sea‐ buckthorn leaf nutrients was relatively less. In some studies, the drought tolerance of germplasm resources was evaluated in terms of growth characteristics, physiological and biochemical traits, and morpho‐anatomical structures using 1‐year‐old seedlings of 10 Chinese sea‐buckthorn species (Li et al. 2020). Previous studies used the affiliation function method to comprehensively evaluate the nutrients of sea‐buckthorn seeds and seedlings from different origins (Slawinska et al. 2023). Previous researchers evaluated the quality of sea‐buckthorn resources in terms of flavonoid content of rutin, isoquercitrin, silymarin, isorhamnetin‐3‐O‐glucoside, quercetin, kaempferol, and isorhamnetin in sea‐buckthorn leaves (Liu et al. 2022). Different methods can screen out good varieties to a certain extent, but each method has its own advantages and limitations and does not intuitively reflect the differences in overall quality among varieties. Therefore, there is an urgent need for scientific and reliable evaluation methods to systematically evaluate the quality of sea‐buckthorn leaves in order to select high‐quality sea ‐buckthorn varieties.

Through systematic research on the quality characteristics of sea‐buckthorn leaves, it provides a theoretical basis for the product development and processing utilization of the sea‐buckthorn leaves, which can promote the expansion of sea‐buckthorn cultivation area to a certain extent, promote the industrialized production of sea‐buckthorn, and achieve higher economic benefits and social value. In this study, we determined the content of leaf nutrients, total flavonoids, and mineral elements. We used factor, principal component, and cluster analyses to comprehensively evaluate the indexes in the leaves of different varieties of sea‐buckthorn in the cold land and preliminarily screened out high‐quality sea‐buckthorn varieties. The research laid a theoretical foundation for the development, processing and utilization of sea‐buckthorn leaves and promoted the development of the industrialization of sea‐buckthorn leaves.

## 2 Materials and Methods

### 2.1 Experimental Design and Management

With ‘201305’, ‘5–3’, ‘TF1‐12’, ‘ck1’, ‘2013–28’, ‘shenqiuhong’, ‘TF2‐28’, ‘5–13’, ‘6’, ‘2012‐e’, ‘42’, ‘TF2‐27’, ‘TF2‐16’, ‘HS‐20’, ‘xiongxingxi‐1’, ‘xiongxingxi‐2’, ‘za‐1‐2’, ‘36’, ‘za54’, ‘fenlan’, ‘xiaolajiao’, ‘juren’, ‘wanhuang’, ‘201306’, ‘201308’, ‘201307’, 26 varieties (strains) were used as the subjects of the study. These varieties (strains) are hybrids with excellent characters and are cultivated over a large area in the region, as shown in Table 1. Sea‐buckthorn leaves were all harvested from the Berry Research Institute of Heilongjiang Provincial Academy of Agricultural Sciences in mid‐July 2018, washed clean with distilled water, and then quickly placed in an insulated box with dry ice to be brought back to the laboratory and kept in a −80°C cryogenic refrigerator for the experiment. Sea‐buckthorn leaves were dried at 80°C (Melike and Zekai 2018) until constant weight, ground into powder, sieved, and kept in a desiccator for reserve.

**TABLE 1:** Sea‐buckthorn strains (varieties).

No.	Strains (varieties)
S1	201,305 (*Hippophae rhamnoides subsp. Mongolica* ⊗)
S2	5‐3 (*Hippophae rhamnoides subsp. sinensis Rousi* × *Hippophae rhamnoides subsp. mongolica*)
S3	TF1‐12 (*Hippophae rhamnoides subsp. sinensis Rousi* × *Hippophae rhamnoides subsp. mongolica*)
S4	ck1 (*Hippophae rhamnoides subsp. sinensis Rousi* × *Hippophae rhamnoides subsp. mongolica*)
S5	2013‐28 (*Hippophae rhamnoides subsp. sinensis Rousi* × *Hippophae rhamnoides subsp. mongolica*)
S6	shenqiuhong (*Hippophae rhamnoides subsp. sinensis Rousi* × *Hippophae rhamnoides subsp. mongolica*)
S7	TF2‐28 (*Hippophae rhamnoides subsp. sinensis Rousi* × *Hippophae rhamnoides subsp. mongolica*)
S8	5‐13 (*Hippophae rhamnoides subsp. sinensis Rousi* × *Hippophae rhamnoides subsp. mongolica*)
S9	6 (*Hippophae rhamnoides subsp. Mongolica* ⊗)
S10	2012‐e (*Hippophae rhamnoides subsp. sinensis Rousi* × *Hippophae rhamnoides subsp. mongolica*)
S11	42 (*Hippophae rhamnoides subsp. sinensis Rousi* × *Hippophae rhamnoides subsp. mongolica*)
S12	TF2‐27 (*Hippophae rhamnoides subsp. sinensis Rousi* × *Hippophae rhamnoides subsp. mongolica*)
S13	TF2‐16 (*Hippophae rhamnoides subsp. sinensis Rousi* × *Hippophae rhamnoides subsp. mongolica*)
S14	HS‐20 (*Hippophae rhamnoides subsp. sinensis Rousi* × *Hippophae rhamnoides subsp. mongolica*)
S15	xiongxingxi‐1 (*Hippophae rhamnoides subsp. sinensis Rousi* ⊗)
S16	xiongxingxi‐2 (*Hippophae rhamnoides subsp. sinensis Rousi* ⊗)
S17	za‐1‐2 (*Hippophae rhamnoides subsp. sinensis Rousi* × *Hippophae rhamnoides subsp. mongolica*)
S18	36 (*Hippophae rhamnoides subsp. sinensis Rousi* × *Hippophae rhamnoides subsp. mongolica*)
S19	za‐54 (*Hippophae rhamnoides subsp. sinensis Rousi* × *Hippophae rhamnoides subsp. mongolica*)
S20	xiaolajiao (*Hippophae rhamnoides subsp. Mongolica* ⊗)
S21	fenlan (*Hippophae rhamnoides subsp. Mongolica* ⊗)
S22	juren (*Hippophae rhamnoides subsp. Mongolica* ⊗)
S23	wanhuang (*Hippophae rhamnoides subsp. sinensis Rousi* × *Hippophae rhamnoides subsp. mongolica*)
S24	201,308 (*Hippophae rhamnoides subsp. Mongolica* ⊗)
S25	201,307 (*Hippophae rhamnoides subsp. Mongolica* ⊗)
S26	201,306 (*Hippophae rhamnoides subsp. Mongolica* ⊗)

### 2.2 Sampling and Measurements

#### 2.2.1 Determination of Leaves Flavonol Composition

Leaf flavonol composition was determined with reference to the method of Criste et al. (2020). By preparing standard solutions of quercetin, kaempferol, catechin, quercetin, and isoquercetin with a concentration of 1 μg/mL, and injecting them with gradients of 10, 30, 60, 90, 120, and 150 μL for liquid chromatography, individual standard regression equations were obtained. The results showed a good linear relationship between injection volume and concentration. Subsequently, precision, stability, and recovery rate tests were conducted on sea buckthorn leaf powder. The results indicated normal instrument precision, relative stability of the samples within 1.5 h, with average recovery rates ranging from 89.17% to 104.33% and RSD ranging from 0.58% to 2.78%, demonstrating the applicability of the method for analytical purposes.

#### 2.2.2 Determination of Leaves Nutrients

According to the national standard for food safety, the determination of polysaccharides adopts the method of Liu et al. (2023). A standard curve was created using a 0.10 mg/mL glucose solution, with gradients of 0.20, 0.40, 0.60, 0.80, and 1.00 mL for liquid chromatography. Anthrone‐sulfuric acid reagent was added, and absorbance was measured at 620 nm. The equation obtained, *y* = 12.453*x* − 0.0281, with *R* ^2^ = 0.997, showed a strong linear relationship for quantifying polysaccharides in sea buckthorn leaves. Subsequently, leaf samples from 26 varieties were extracted and analyzed. The polysaccharide content was calculated using the recorded absorbance values.

The content of Vc was determined with reference to the method of Sytarová et al. (2020). Using a standard solution of 2.00 mg/mL VC‐dihydrogen ammonium phosphate, a standard curve was constructed with sample gradients of 10–50 μL. The equation for the curve is *y* = 0.053 + 0.049*x*, with *R* ^2^ = 0.996, making it suitable for quantifying VC in sea buckthorn leaf samples. Liquid chromatography analysis of powdered leaf samples from 26 sea buckthorn varieties indicated normal instrument precision, high sample stability (RSD = 0.86%), a recovery rate of 105.78%, with an RSD of 1.1%.

The content of crude protein was determined with reference to the method of alkaline extraction of sea‐buckthorn leaf protein by Saracila et al. (2022) and the content determined by Kjeldahl nitrogen determination. Precision, stability, and recovery rate tests indicate that the instrument precision is normal (RSD = 2.31%), sample stability is good (RSD = 1.56%), with a recovery rate of 100.26%, and an RSD of 3.00%.

The caffeine content was determined with reference to the method of measuring caffeine content in functional beverages by Xin et al. (2021). The standard curve, based on a 0.20 mg/mL caffeine solution, demonstrated strong linearity (*Y* = 0.124*X* − 0.116, *R* ^2^ = 0.995), suitable for caffeine quantification in sea buckthorn leaves. Caffeine content in various sea buckthorn leaf varieties was determined following established methods. Randomly selected samples underwent precision, stability, and recovery rate tests, showing normal instrument precision (RSD = 1.22%), sample stability within 6 h (RSD = 3.33%), and a 95.14% recovery rate with an RSD of 2.08%, validating the method's analytical reliability.

The content of crude fiber was determined by the improved method by Nour et al. (2021). Precision, stability, and recovery rate tests showed normal instrument precision (RSD = 1.89%), good sample stability (RSD = 0.78%), with a recovery rate of 91.84% and an RSD of 2.23%.

The crude fat mass fraction was determined by the Soxhlet extraction method with reference to Saracila et al. (2022). The results of precision, stability, and recovery showed that the precision of crude fat content was normal (RSD = 0.47%), the sample was relatively stable within 6 h (RSD = 2.96%), the recovery was 104.26%, RSD was 1.05%.

#### 2.2.3 Determination of Leaves Mineral Elements

Determination of K and Na by the method of Tkacz et al. (2021). The content of Ca was determined using the method of Efimova et al. (2022). The content of Mg was determined using the method of Wang et al. (2022). The content of Cu, Mn, Zn, Fe was determined using the method of Dadhwal et al. (2023). In the sample digestion step, 0.50 g of powder from each of the 26 varieties of sea buckthorn leaves was mixed with 10.00 mL of nitric acid and digested using a microwave for 20 min. After digestion, the solution was cooled and made up to 50 mL with deionized water. For constructing the standard curve, standard solutions of Na, K, Ca, Mg, Fe, Mn, Cu, and Zn at 1 μg/mL were prepared and diluted with HNO_3_ to create different gradient solutions. The resulting curve allows for the quantitative analysis of these elements in sea buckthorn leaves.

#### 2.2.4 Determination of Leaves Secondary Metabolites

The determination of total polyphenols and total flavonoids was referred to the method of Kreps et al. (2021). A standard curve was prepared using a rutin solution (0.20 mg/mL), treated sequentially with NaNO_2_, Al(NO_3_)_3_, NaOH, and methanol. The equation *y* = 12.453*x* − 0.0281 (*R* ^2^ = 0.997) demonstrated a strong linear relationship for flavonoid quantification in sea buckthorn leaves. Next, extracts from 26 sea buckthorn varieties were processed at 50°C for 60 min, with subsequent absorbance measurement. Evaluation indicated satisfactory instrument precision, sample stability up to 3 h, and a flavonoid recovery rate of 93.5%.

A standard curve was created using gallic acid solution (0.02 mg/mL), followed by the addition of Folin–Ciocalteu reagent and Na_2_CO_3_ solution. After dilution with 50% ethanol and incubation, absorbance was measured at 735 nm. The resulting equation (*y* = 9.705*x* + 0.0417, *R* ^2^ = 0.9993) showed strong linearity for polyphenol quantification in sea buckthorn leaves. Sample analysis involved extracting 1.00 g of powdered leaves from 26 varieties with 50% ethanol under ultrasonic conditions (350 W, 80°C), followed by dilution. Precision, stability, and recovery rate tests yielded satisfactory results (RSD = 0.78%, 1.48%, and 1.57%, respectively), confirming the method's suitability for analysis.

### 2.3 Statistical Analysis

Peakview 2.2 and Masterview 2.0 were used to analyze the data measured by ultra‐performance liquid chromatography‐mass spectrometry (UPLC‐MS); All experimental data were analyzed mathematically (factor analysis, principal component analysis, cluster analysis) using IBM SPSS Statistics 22; Excel 2016 was applied for tabulation and Origin 8.0 for plotting.

## 3 Results

### 3.1 Analysis of Flavonol Composition and Content in Different Sea‐Buckthorn Leaves

**FIGURE 1:**
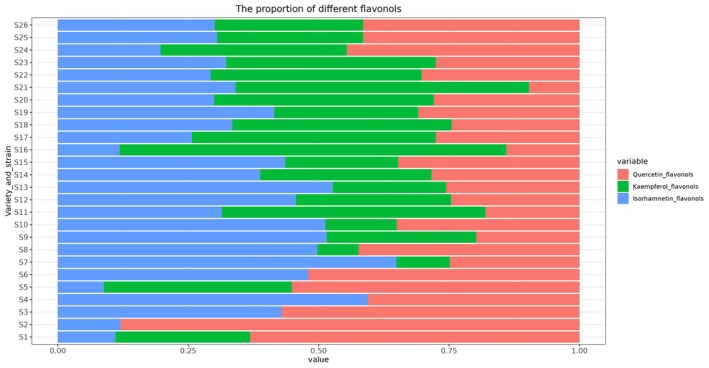
The flavonol composition in different sea‐buckthorn leaves.

A total of 14 flavonol substances were identified by UHPLC–MS analysis in the leaves of different varieties (strains) of sea‐buckthorn. The 14 flavonol substances belonged to the three major classes of isorhamnetin, kaempferol, and quercetin flavonols, respectively. The relative contents of isorhamnetin flavonols, kaempferol flavonols, and quercetin flavonols in the leaves of 26 sea‐buckthorn varieties (strains) are presented in Figure 1.

From the perspective of isorhamnetin flavonols content, ‘S7’, ‘S4’, ‘S13’, ‘S9’, ‘S10’, ‘S8’, ‘S6’, ‘S12’, ‘S12’, ‘S3’, ‘S19’ leaves had relatively high isorhamnetin flavonols content (above 40%). ‘S2’, ‘S16’, ‘S1’, ‘S5’ leaves had relatively low isorhamnetin flavonols content (less than 20%), basically at the lowest level. From the perspective of kaempferol flavonols content, ‘S16’, ‘S21’, ‘S11’, ‘S17’, ‘S20’, ‘S18’, ‘S22’, ‘S23’ leaves had relatively high kaempferol flavonols content (above 40%). ‘S10’, ‘S7’, ‘S8’ leaves had relatively low kaempferol flavonols content (less than 20%), basically at the lowest level. From the perspective of quercetin flavonols content, ‘S2’, ‘S3’, ‘S5’, ‘S6’, ‘S24’, ‘S8’, ‘S25’, ‘S26’ and ‘S4’ leaves had relatively high quercetin flavonol content (above 40%). ‘S9’, ‘S11’, ‘S16’ and ‘S21’ leaves had relatively low quercetin flavonol content (less than 20%), basically at the lowest level.

Overall, there are relatively obvious differences in the content of the three flavonols in the leaves of different sea buckthorn varieties (strains). The flavonol contents in the leaves of the 26 sea‐buckthorn varieties (strains) were characterized by the following basic features: relatively high contents of isorhamnetin flavonols and quercetin flavonols and low contents of kaempferol flavonols.

**FIGURE 2:**
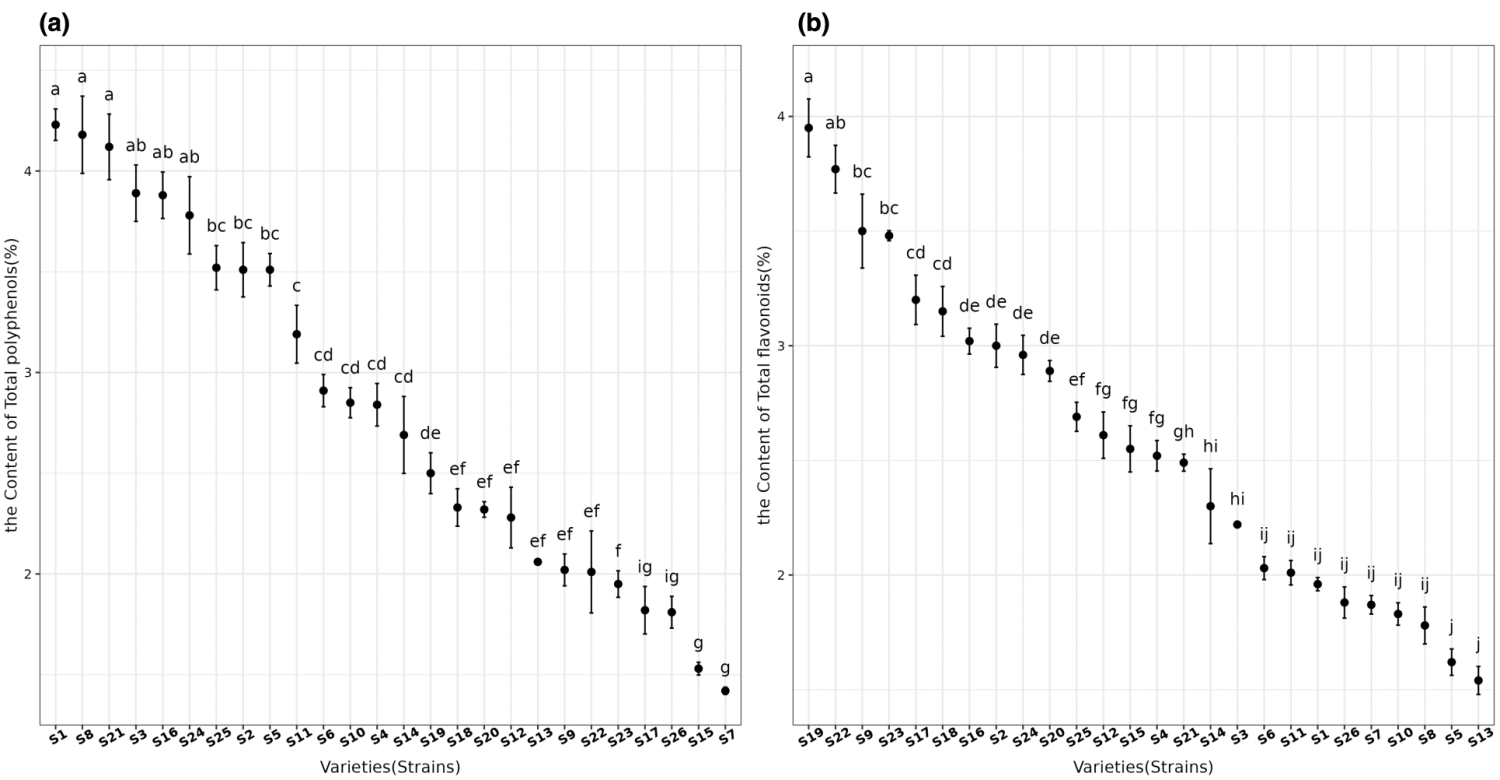
The content of secondary metabolic substances in the leaves of sea‐buckthorn of different varieties (strains).

The content of secondary metabolic substances in the leaves of sea buckthorn of different varieties (strains) is shown in Figure 2. As can be seen from Figure 2b, the total flavonoid content in ‘S19’ and ‘S22’ leaves were the highest (higher than 3.5%). The total flavonoids content in ‘S1’, ‘S26’, ‘S7’, ‘S10’, ‘S8’, ‘S5’ and ‘S13’ leaves was lower than that 2%, which were significantly lower than that of other sea‐buckthorn varieties (strains). As can be seen from Figure 2a, the total flavonoids content in ‘S1’, ‘S8’ and ‘S21’ leaves was the highest (higher than 4%). The total flavonoids content in ‘S23’, ‘S17’, ‘S26’, ‘S15’ and ‘S7’ leaves was lower than 2%, which was significantly lower than that of other sea‐buckthorn varieties (strains).

### 3.2 Analysis of Main Nutrients in Different Sea‐Buckthorn Leaves

**FIGURE 3:**
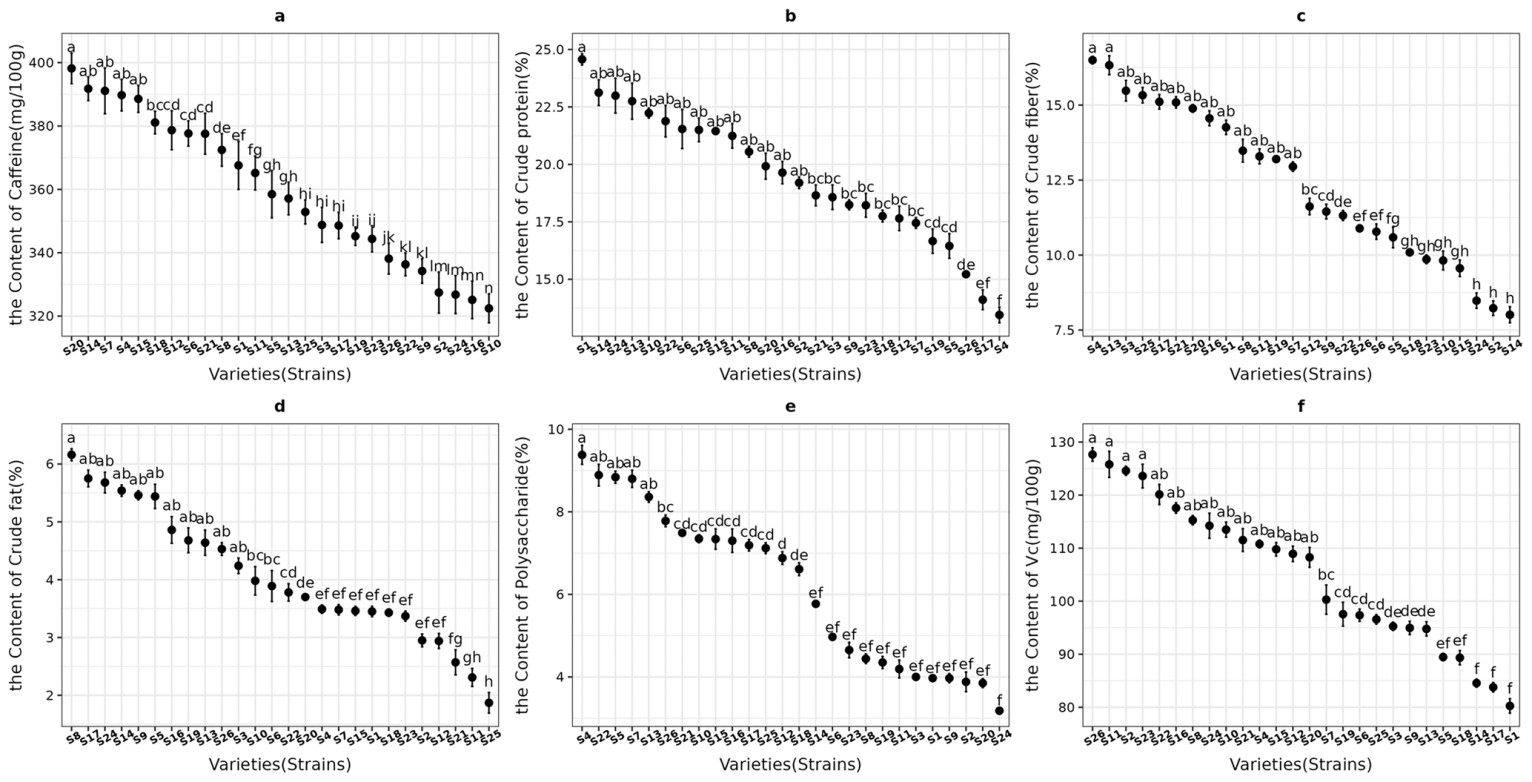
The content of nutrients in the leaves of sea buckthorn of different varieties (strains).

The content of nutrients in the leaves of sea buckthorn of different varieties (strains) is shown in Figure 3. As can be seen from Figure 3a, the caffeine content of all varieties (strains) is above 325 mg/100 g. As can be seen from Figure 3b, the crude protein content in ‘S1’ leaves was the highest (higher than 23%). The crude protein content of ‘S17’ and ‘S4’ leaves was lower than 15%, which was significantly lower than that of other sea‐buckthorn varieties (strains). As can be seen from Figure 3c, the crude fiber content in ‘S4’ and ‘S13’ leaves was the highest (higher than 16%). The crude fiber content of ‘S24’, ‘S2’ and ‘S14’ leaves was lower than 9%, which was significantly lower than that of other sea‐buckthorn varieties (strains). As can be seen from Figure 3d, the crude fat content in ‘S8’ leaves was the highest (higher than 6%). The crude fat content of ‘S21’, ‘S11’ and ‘S25’ leaves was lower than 3%, which was significantly lower than that of other sea‐buckthorn varieties (strains). As can be seen from Figure 3e, the polysaccharide content in ‘S4’, ‘S22’, ‘S5’ and ‘S7’ leaves was the highest (higher than 8%). The polysaccharide content of ‘S24’ leaves was lower than 4%, which was significantly lower than that of other sea‐buckthorn varieties (strains). As can be seen from Figure 3f, the Vc content of all varieties (strains) is above 80 mg/100 g.

### 3.3 Analysis of Mineral Elements in Different Sea‐Buckthorn Leaves

The content measurement results of Na, K, Ca, Mg, Fe, Mn, Cu, and Zn in the leaves of 26 sea‐buckthorn varieties (strains) are shown in Table 2. It can be seen from the table that the mineral elements in sea‐buckthorn leaves have the following characteristics: The Na content in ‘S14’ leaves was the highest (higher than 45 mg/100 g). The Na content of ‘S1’ leaves was lower than 30 mg/100 g, which was significantly lower than that of other sea‐buckthorn varieties (strains). The K content in ‘S8’, ‘S2’, ‘S3’, ‘S5’, ‘S6’, ‘S9’, ‘S13’, ‘S14’, ‘S16’ and ‘S18’ leaves was the highest (higher than 21 mg/100 g). The K content of ‘S26’ leaves was lower than 13 mg/100 g, which was significantly lower than that of other sea‐buckthorn varieties (strains). The Ca content in ‘S4’, ‘S6’, ‘S9’, ‘S10’, ‘S13’, ‘S15’, ‘S16’ leaves was the highest (higher than 140 mg/100 g). The K content of ‘S11’ leaves was lower than 117 mg/100 g, which was significantly lower than that of other sea‐buckthorn varieties (strains). The Mg content in ‘S26’ leaves was the highest (higher than 360 mg/100 g). The Mg content of ‘S6’ leaves was lower than 120 mg/100 g, which was significantly lower than that of other sea‐buckthorn varieties (strains).

**TABLE 2:** Mineral elements content in leaves of different seabuckthorn varieties (strains).

Varieties (strains)	Na	K	Ca	Mg	Fe	Mn	Cu	Zn
S1	25.67 ± 0.354b	18.36 ± 0.152cd	134.15 ± 1.84ab	149.31 ± 0.48jk	41.93 ± 0.647ab	25.93 ± 0.005gh	4.67 ± 0.059bc	10.78 ± 0.244bc
S2	36.95 ± 0.459ab	25.25 ± 0.465ab	129.19 ± 3.7bc	166.8 ± 0.25i	33.24 ± 0.852cd	23.91 ± 0.003gh	4.04 ± 0.099bc	14.57 ± 0.344ab
S3	40.79 ± 0.358a	23.17 ± 0.384ab	139.82 ± 6.26a	130.16 ± 0.416mn	46.81 ± 0.952ab	45.87 ± 0.013de	7 ± 0.049a	9.39 ± 0.295cd
S4	41.5 ± 0.458a	20.89 ± 0.192bc	141.15 ± 7.75a	153.63 ± 0.908jk	30.22 ± 0.753de	41.84 ± 0.005de	3.7 ± 0.068cd	12.81 ± 0.294bc
S5	42.55 ± 0.486a	23.84 ± 0.446ab	134.79 ± 4.955ab	147.55 ± 0.722kl	44.88 ± 0.741ab	47.97 ± 0.007d	4.24 ± 0.079bc	14.06 ± 0.393ab
S6	41.69 ± 0.457a	21.07 ± 0.107ab	141.26 ± 6.471a	118.01 ± 0.401o	47.52 ± 0.951ab	50.39 ± 0.003ce	4 ± 8560bc	11.79 ± 0.428bc
S7	41.46 ± 0.258a	20.53 ± 0.409bc	139.8 ± 5.055a	135.56 ± 0.238mn	43.77 ± 0.753ab	39.25 ± 0.003de	2.48 ± 0.049ef	9.21 ± 0.393cd
S8	43.78 ± 0.287a	26.76 ± 0.415a	134.8 ± 5.83ab	158.33 ± 0.26ij	39.56 ± 0.852bc	32.69 ± 0.008fg	3.57 ± 0.069cd	13.19 ± 0.479bc
S9	41.1 ± 0.255a	22.06 ± 0.316ab	141.5 ± 3.776a	125.8 ± 0.286no	46.89 ± 0.963ab	88.95 ± 0.008a	3.17 ± 0.059de	24.09 ± 0.434ab
S10	44.28 ± 1.54a	20.7 ± 4.75bc	140.73 ± 1.55a	127.16 ± 0.26no	53.2 ± 0.475ab	72.55 ± 0.008b	5.07 ± 0.07b	27.43 ± 0.393a
S11	41.81 ± 0.374a	19.4 ± 0.36bc	116.04 ± 4.903g	333.46 ± 1.048b	25.09 ± 0.265ef	14.42 ± 0.009jk	2.9 ± 0.058de	17.49 ± 0.448ab
S12	43.58 ± 0.457a	20.24 ± 0.364bc	137.86 ± 7.66ab	160.49 ± 0.94ij	46.67 ± 0.159ab	58.57 ± 0.008c	4.17 ± 0.048bc	17.43 ± 0.299ab
S13	42.07 ± 0.485a	24.41 ± 0.151ab	141.17 ± 3.23a	158.69 ± 0.808ij	47.14 ± 0.753ab	46.8 ± 0.006de	2.29 ± 0.059fg	14.48 ± 0.089ab
S14	45.64 ± 0.157a	23.8 ± 0.271ab	139.9 ± 2.39a	135.93 ± 0.26mn	48.79 ± 0.854ab	47.32 ± 0.009de	3.28 ± 0.07de	23.45 ± 0.735ab
S15	42.12 ± 0.464a	21.81 ± 0.214ab	141.42 ± 4.57a	140.12 ± 0.332lm	46.05 ± 0.964ab	37.67 ± 0.01de	4.03 ± 0.078bc	17.92 ± 0.127ab
S16	43.8 ± 0.267a	22.39 ± 0.526ab	142.2 ± 1.859a	131.92 ± 0.518mn	54.2 ± 0.854a	31.4 ± 0.01 fg	3.74 ± 0.059ed	20.55 ± 0.432ab
S17	42.02 ± 0.489a	20.31 ± 0.262bc	124.32 ± 4.05de	295.59 ± 0.822c	19.2 ± 0.952fg	18.2 ± 0.005gk	2.65 ± 0.099ef	7.92 ± 0.129d
S18	42.27 ± 0.478a	21.05 ± 0.41ab	123.9 ± 3.92de	247.67 ± 0.268e	21.57 ± 0.574ef	14.69 ± 0.006gk	2.16 ± 0.079fg	8.48 ± 0.074d
S19	40.92 ± 0.368a	19.36 ± 0.375bc	127.07 ± 3.82cd	332.09 ± 1.104b	18.9 ± 0.234fg	19.17 ± 0.005ij	2 ± 0.178fg	7.31 ± 0.129d
S20	40.52 ± 0.468a	15.69 ± 0.19cd	128.38 ± 2.23bc	199.88 ± 0.338h	31.83 ± 0.864cd	25.57 ± 0.015gh	1.94 ± 0.089fg	7.76 ± 0.185d
S21	41.48 ± 0.369a	16.2 ± 0.445cd	121.09 ± 5.15ef	227.05 ± 1.348f	18.93 ± 0.741fg	21.01 ± 0.003hi	1.51 ± 0.076gh	6.86 ± 0.477d
S22	43.56 ± 0.347a	18.53 ± 0.232cd	134.63 ± 1.39ab	215.57 ± 0.956g	21.68 ± 0.789ef	19.8 ± 0.005ij	1.81 ± 0.069gh	15.97 ± 0.377ab
S23	40.11 ± 0.358a	15.78 ± 0.312cd	132.63 ± 4.26ab	249.37 ± 1.404e	26.63 ± 0.854ef	34.87 ± 0.019ef	2.25 ± 0.06fg	7.65 ± 0.443d
S24	41.67 ± 0.385a	16.54 ± 0.55cd	119.19 ± 2.23fg	301.38 ± 1.388c	32.75 ± 0.957cd	12.44 ± 0.006jk	1.59 ± 0.098gh	8.58 ± 0.477d
S25	39.96 ± 0.469a	15.16 ± 0.406cd	127.68 ± 2.47cd	284.06 ± 1.29d	16.38 ± 0.759g	14.76 ± 0.006jk	1.63 ± 0.065gh	7.79 ± 0.476d
S26	35.91 ± 0.358ab	12.88 ± 0.506d	129.63 ± 6.65bc	364.16 ± 0.956a	22.17 ± 0.859ef	9.14 ± 0.006k	0.8 ± 0.059h	7.84 ± 0.376d

Meanwhile, Fe content in ‘S16’ leaves was the highest (higher than 50 mg/100 g). The Fe content of ‘S25’ leaves was lower than 17 mg/100 g, which was significantly lower than that of other sea‐buckthorn varieties (strains). The Mn content in ‘S9’ leaves was the highest (higher than 85 mg/100 g). The Mn content of ‘S26’ leaves was lower than 10 mg/100 g, which was significantly lower than that of other sea‐buckthorn varieties (strains). The Cu content in ‘S3’ leaves was the highest (higher than 6 mg/100 g). The Cu content of ‘S26’ leaves was lower than 1 mg/100 g, which was significantly lower than that of other sea buckthorn varieties (strains). The Zn content in ‘S10’ leaves was the highest (higher than 25 mg/100 g). The Zn content of ‘S21’ leaves was lower than 7 mg/100 g, which was significantly lower than that of other sea‐buckthorn varieties (strains).

### 3.4 Preliminary Evaluation of Quality of Sea‐Buckthorn Leaves

#### 3.4.1 Factor Analysis of Sea‐Buckthorn Leaves

Factor analysis was performed on the leaves of 26 sea‐buckthorn varieties (strains), and common factors were extracted by the principal component extraction method. The analysis results are shown in Tables 3 and 4. As can be seen from Table 3, there are five common factors with eigenvalues greater than 1 (λ > 1). The first common factor has a large load on Fe content, Ca content, and Mn content. The second common factor has a large load on total polyphenol content. The third common factor has a large load on crude fiber content and polysaccharide content. The fourth common factor has a large load on crude fat content, and the fifth common factor has a large load on Vc content. The cumulative contribution rate of these five common factors was 75.344%, which was sufficient to describe the nutritional status of sea‐buckthorn leaves.

**TABLE 3:** Factor load and cumulative contribution rate in sea buckthorn leaves nutrition quality.

Nutrient content	Principal component				
	1	2	3	4	5
Total flavonoids	−0.798	0.365	−0.063	−0.084	−0.009
Total polyphenols	0.035	0.875	0.044	0.035	0.146
Polysaccharide	0.175	−0.583	0.513	−0.185	0.009
*V*_C_	−0.155	−0.141	−0.068	−0.333	0.793
Caffeine	0.06	−0.202	0.07	−0.287	−0.767
Crude protein	0.177	0.361	−0.715	−0.209	−0.078
Crude fiber	−0.059	0.282	0.746	−0.158	−0.234
Crude fat	0.15	−0.032	−0.05	0.908	−0.017
Na	0.194	−0.501	0.007	0.355	0.184
K	0.692	0.107	0.039	0.439	−0.066
Ca	0.874	−0.2	0.088	−0.01	−0.101
Mg	−0.922	−0.077	0.026	0.042	0.214
Fe	0.895	0.078	−0.222	0.121	−0.058
Mn	0.826	−0.165	−0.092	0.135	−0.034
Cu	0.768	0.427	0.065	0.04	−0.016
Zn	0.714	−0.162	−0.355	0.089	0.301
The eigenvalue	5.467	2.061	1.547	1.492	1.489
Rate of contribution (%)	34.166	12.884	9.666	9.322	9.306
The cumulative contribution rate (%)	35.708	49.323	60.634	68.728	75.344

**TABLE 4:** Factor score and comprehensive score in nutrient of sea‐buckthorn leaves.

Varieties (strains)	Factor 1 score	Factor 2 score	Factor 3 score	Factor 4 score	Factor 5 score	Comprehensive scores	Rank
S3	0.945	2.087	1.161	0.465	−0.110	0.978	1
S16	1.094	0.672	0.870	0.363	1.692	0.976	2
S10	1.673	−0.276	−0.817	−0.310	1.707	0.779	3
S8	0.473	0.944	0.247	1.496	0.188	0.616	4
S5	0.739	−0.084	0.834	1.336	−0.194	0.569	5
S9	1.111	−0.469	−0.676	1.238	0.205	0.515	6
S4	0.630	−0.493	2.470	−0.748	−0.068	0.417	7
S2	0.341	0.927	−0.568	−0.378	1.623	0.394	8
S13	0.749	−0.448	0.438	0.226	−0.665	0.265	9
S12	0.956	−0.829	0.082	−0.865	0.021	0.198	10
S1	0.264	2.690	−0.567	−1.299	−1.731	0.132	11
S6	0.691	0.201	−0.811	−0.251	−0.950	0.095	12
S15	0.955	−1.281	−0.954	−0.979	−0.521	−0.094	13
S14	0.657	−0.650	−2.021	1.157	−1.457	−0.109	14
S7	0.511	−1.513	0.613	−0.751	−1.153	−0.184	15
S17	−1.118	−0.564	1.559	2.012	−0.628	−0.232	16
S22	−0.395	−1.074	−0.313	−0.554	1.103	−0.335	17
S19	−1.374	0.067	0.371	1.227	−0.125	−0.427	18
S11	−0.995	0.589	−0.659	−0.983	0.965	−0.437	19
S21	−1.149	0.609	0.872	−1.097	−0.067	−0.449	20
S23	−0.639	−0.696	−0.538	−0.628	0.802	−0.456	21
S24	−1.595	0.857	−1.825	1.361	0.815	−0.542	22
S25	−1.124	0.545	0.424	−1.392	−0.240	−0.564	23
S20	−0.800	−0.047	−0.344	−0.712	−1.249	−0.657	24
S26	−1.554	−1.123	0.428	−0.251	1.225	−0.722	25
S18	−1.046	−0.638	−0.277	0.317	−1.185	−0.726	26

With *Y*_1_, *Y*_2_, *Y*_3_, *Y*_4_, *Y*_5_, *Y*_6_, *Y*_7_, *Y*_8_, *Y*_9_, *Y*_10_, *Y*_11_, *Y*_12_, *Y*_13_, *Y*_14_, *Y*_15_, *Y*_16_ representing 16 nutrition indices, the factor score to the five common factor coefficient matrix are expressed as the linear equation of 16 indicators. The factor score function is as follows:

$$\begin{array}{cc} F_{1}=\; & -0.15^{*}Y_{1}+0.049^{*}Y_{2}+0.07^{*}Y_{3}+0.047^{*}Y_{4}-0.01^{*}Y_{5}\\ & +0.021^{*}Y_{6}+0.049^{*}Y_{7}-0.074^{*}Y_{8}-0.004^{*}Y_{9}+0.1^{*}Y_{10}\\ & +0.177^{*}Y_{11}-0.185^{*}Y_{12}+0.159^{*}Y_{13}+0.145^{*}Y_{14}\\ & +0.176^{*}Y_{15}+0.131^{*}Y_{16} \end{array}$$

$$\begin{array}{cc} F_{2}=\; & 0.147^{*}Y_{1}+0.46^{*}Y_{2}-0.235^{*}Y_{3}-0.043^{*}Y_{4}-0.142^{*}Y_{5}\\ & +0.102^{*}Y_{6}+0.208^{*}Y_{7}-0.003^{*}Y_{8}-0.23^{*}Y_{9}+0.087^{*}Y_{10}\\ & -0.066^{*}Y_{11}-0.057^{*}Y_{12}+0.046^{*}Y_{13}-0.064^{*}Y_{14}\\ & +0.252^{*}Y_{15}-0.077^{*}Y_{16} \end{array}$$

$$\begin{array}{cc} F_{3}= & -0.062^{*}Y_{1}+0.158^{*}Y_{2}+0.312^{*}Y_{3}\\ & +0.048^{*}Y_{4}-0.108^{*}Y_{5}-0.484^{*}Y_{6}\\ & +0.521^{*}Y_{7}-0.001^{*}Y_{8}+0.006^{*}Y_{9}\\ & +0.09^{*}Y_{10}+0.081^{*}Y_{11}-0.013^{*}Y_{12}\\ & -0.098^{*}Y_{13}-0.031^{*}Y_{14}+0.144^{*}Y_{15}-0.173^{*}Y_{16} \end{array}$$

$$\begin{array}{cc} F_{4}= & 0.029^{*}Y_{1}+0.03^{*}Y_{2}-0.147^{*}Y_{3}-0.269^{*}Y_{4}\\ & -0.183^{*}Y_{5}-0.188^{*}Y_{6}-0.07^{*}Y_{7}+0.651^{*}Y_{8}\\ & +0.224^{*}Y_{9}+0.254^{*}Y_{10}-0.099^{*}Y_{11}+0.122^{*}Y_{12}\\ & -0.013^{*}Y_{13}+0.005^{*}Y_{14}-0.045^{*}Y_{15}-0.041^{*}Y_{16} \end{array}$$

$$\begin{array}{cc} F_{5}= & -0.045^{*}Y_{1}+0.166^{*}Y_{2}+0.08^{*}Y_{3}+0.561^{*}Y_{4}-0.541^{*}Y_{5}\\ & -0.136^{*}Y_{6}-0.023^{*}Y_{7}-0.054^{*}Y_{8}+0.102^{*}Y_{9}-0.009^{*}Y_{10}\\ & -0.011^{*}Y_{11}+0.092^{*}Y_{12}-0.021^{*}Y_{13}-0.001^{*}Y_{14}+0.074^{*}Y_{15}+0.193^{*}Y_{16} \end{array}$$

The above five common factors reflect the nutritional status of sea‐buckthorn leaves from different aspects, but a single common factor cannot fully describe the quality of sea‐buckthorn leaves. Therefore, the variance contribution rate of each common factor is used as the weight coefficient to calculate the comprehensive statistics, and the formula is as follows:

$$F=0.453^{*}F_{1}+0.171^{*}F_{2}+0.128^{*}F_{3}+0.124^{*}F_{4}+0.124^{*}F_{5}$$

The composite scores and five common factor scores of leaves of different sea buckthorn varieties (strains) were calculated by the formula (Table 4). As can be seen from Table 4, the sea‐buckthorn variety (strain) that ranked first in factor 1 score was ‘S10’. ‘S1’ ranked first in factor 2 score. The sea‐buckthorn variety (strain) ranked first in factor 3 score was ‘S4’. The sea‐buckthorn variety (strain) ranked first in factor 4 score was ‘S17’. The sea‐buckthorn variety (strain) ranked first in factor 5 score was ‘S10’. The top scores in factor analysis were ‘S3’, ‘S16’, ‘S10’, ‘S8’, ‘S5’ and ‘S9’, and their comprehensive scores were all over 0.5, among which ‘S3’ was obviously superior to other varieties (strains) and had the highest comprehensive score.

#### 3.4.2 Principal Component Analysis of Sea‐Buckthorn Leaves

Principal component analysis was performed on the leaves of 26 sea‐buckthorn varieties (strains). As can be seen from Table 5, the variance of the first principal component accounted for 35.708% of the total variance. The variance of the second principal component accounted for 13.615%, the variance of the third principal component accounted for 11.311%, the variance of the fourth principal component accounted for 8.094%, and the variance of the fifth principal component accounted for 6.616%. The cumulative variance contribution of these five principal components was 75.344%, which was sufficient to describe the characteristics of sea‐buckthorn varieties (strains).

**TABLE 5:** Composition matrix and explanation of total variance in nutrients of sea‐buckthorn leaves.

Nutrient content	Principal component				
	1	2	3	4	5
Total flavonoids	−0.812	0.332	0.055	0.075	−0.06
Total polyphenols	−0.036	0.774	0.209	0.115	0.367
Polysaccharide	0.127	−0.749	0.128	−0.14	0.237
*V*_C_	−0.249	−0.041	−0.588	−0.418	0.451
Caffeine	0.061	−0.283	0.607	−0.189	−0.482
Crude protein	0.179	0.631	−0.129	−0.353	−0.389
Crude fiber	−0.164	−0.107	0.697	0.104	0.429
Crude fat	0.328	0.023	−0.248	0.817	−0.117
Na	0.281	−0.419	−0.378	0.227	−0.013
K	0.75	0.102	0.046	0.33	0.073
Ca	0.861	−0.206	0.124	−0.139	0.069
Mg	−0.898	−0.082	−0.253	0.161	−0.003
Fe	0.912	0.188	−0.009	−0.07	−0.044
Mn	0.852	−0.086	−0.049	−0.043	−0.019
Cu	0.717	0.369	0.243	−0.023	0.26
Zn	0.737	0.058	−0.429	−0.174	0.015
The eigenvalue	5.713	2.178	1.81	1.295	1.059
Rate of contribution (%)	35.708	13.615	11.311	8.094	6.616
The cumulative contribution rate (%)	35.708	49.323	60.634	68.728	75.344

The coefficient of Fe, Ca, and Mn content in the first principal component is the largest; The coefficient of total polyphenol content in the second principal component was the largest. In the third principal component, the coefficient of crude fiber content is the largest. The coefficient of crude fat content in the fourth principal component is the largest. The coefficient of VC content in the fifth principal component is the largest. These results indicated that the main factors affecting the nutrient composition of sea‐buckthorn leaves were (1) Fe, Ca, and Mn content; (2) total polyphenol content; (3) crude fiber content; (4) crude fat content; (5) VC content. Considering that the cumulative contribution rate of the first five principal components has reached more than 75%, which aggregates relatively more information about the nutrient composition in sea‐buckthorn leaves, it is possible to make a more accurate evaluation of the nutrient composition of sea‐buckthorn leaves from these five aspects.

*X*_1_, *X*_2_, *X*_3_, *X*_4_, and *X*_5_ used to represent the five principal component scores successively. The weight of the five principal components is the ratio of the sum of their respective feature roots and the total feature roots. *F* represents the comprehensive score of the principal component, and the calculation formula of the principal component synthesis is as follows:

$$F=0.47^{*}X_{1}+0.18^{*}X_{2}+0.15^{*}X_{3}+0.11^{*}X_{4}+0.09^{*}X_{5}$$

Obtained from Table 4, sea‐buckthorn varieties (strains) by factor analysis to get the top 10 comprehensive scores from high to low in turn for ‘S3’, ‘S16’, ‘S10’, ‘S8’, ‘S5’, ‘S9’, ‘S4’, ‘S2’, ‘S13’, ‘S12’. As can be seen from Table 6, the comprehensive scores of the top ten companies obtained through principal component analysis are as follows, in order from high to low: ‘S3’, ‘S10’, ‘S9’, ‘S16’, ‘S5’, ‘S14’, ‘S8’, ‘S6’, ‘S1’, ‘S13’. Comparing the top ten comprehensive rankings of the principal component analysis and the top ten comprehensive rankings of the factor analysis, the results are roughly the same, indicating that the results have a certain credibility.

**TABLE 6:** Principal component score and comprehensive score in nutrients of sea‐buckthorn leaves.

Varieties (strains)	Principal component 1 score	Principal component 2 score	Principal component 3 score	Principal component 4 score	Principal component 5 score	Comprehensive scores	Rank
S3	1.796991	2.019019	1.827231	0.923153	1.412151	1.713983	1
S10	3.713875	0.431348	−2.09208	−1.14462	0.812446	1.472352	2
S9	3.353004	−0.05509	−1.21001	0.844678	−0.40906	1.452297	3
S16	2.129873	0.577645	−0.74252	0.276427	2.080753	1.214724	4
S5	2.184775	−0.59915	0.356656	1.546527	0.404561	1.182363	5
S14	2.855367	0.363328	−0.63848	0.674515	−2.59013	1.168068	6
S8	1.53615	1.182961	−0.04827	1.674277	0.49053	1.157492	7
S6	1.786642	0.689835	0.608992	−0.46535	−1.02425	0.922887	8
S1	−0.05583	3.643393	3.008271	−1.02434	−0.65725	0.915787	9
S13	1.924986	−0.92499	0.760278	0.266105	−0.18403	0.871709	10
TF2‐27	1.946928	−1.14645	0.063932	−1.18472	0.070451	0.604031	11
S12	2.289992	−1.13271	−0.30881	−1.57758	−1.13333	0.565233	12
S4	0.656059	−2.29091	1.926142	−0.27408	1.695436	0.305519	13
S2	0.334005	1.790196	−1.4018	−0.7422	1.065352	0.285169	14
S7	1.145163	−2.55164	1.191251	−0.75019	−0.6363	0.123981	15
S17	−1.83225	−1.79477	0.641913	2.818323	0.123911	−0.78266	16
S20	−1.90443	−0.05155	1.12175	−0.54237	−1.15977	−0.90358	17
S22	−1.08309	−1.1053	−1.64497	−0.89584	0.230462	−1.036	18
S18	−1.92704	−0.82697	0.449889	0.542157	−1.37261	−1.05746	19
S19	−2.70378	−0.1655	−0.15944	1.69748	−0.1411	−1.16526	20
S23	−1.65301	−0.50958	−1.33642	−0.90435	−0.05559	−1.17815	21
S24	−2.94098	2.432968	−2.3514	1.228962	−0.84444	−1.24926	22
S11	−2.86588	1.251232	−0.90831	−1.11796	0.312407	−1.36114	23
S21	−3.46654	0.131111	1.224011	−0.63292	0.658959	−1.44557	24
S25	−3.40432	0.308738	1.152101	−1.06385	0.244941	−1.47743	25
S26	−3.81663	−1.66713	−1.48992	−0.1722	0.605519	−2.29907	26

#### 3.4.3 Cluster Analysis of Sea‐Buckthorn Leaves

**FIGURE 4:**
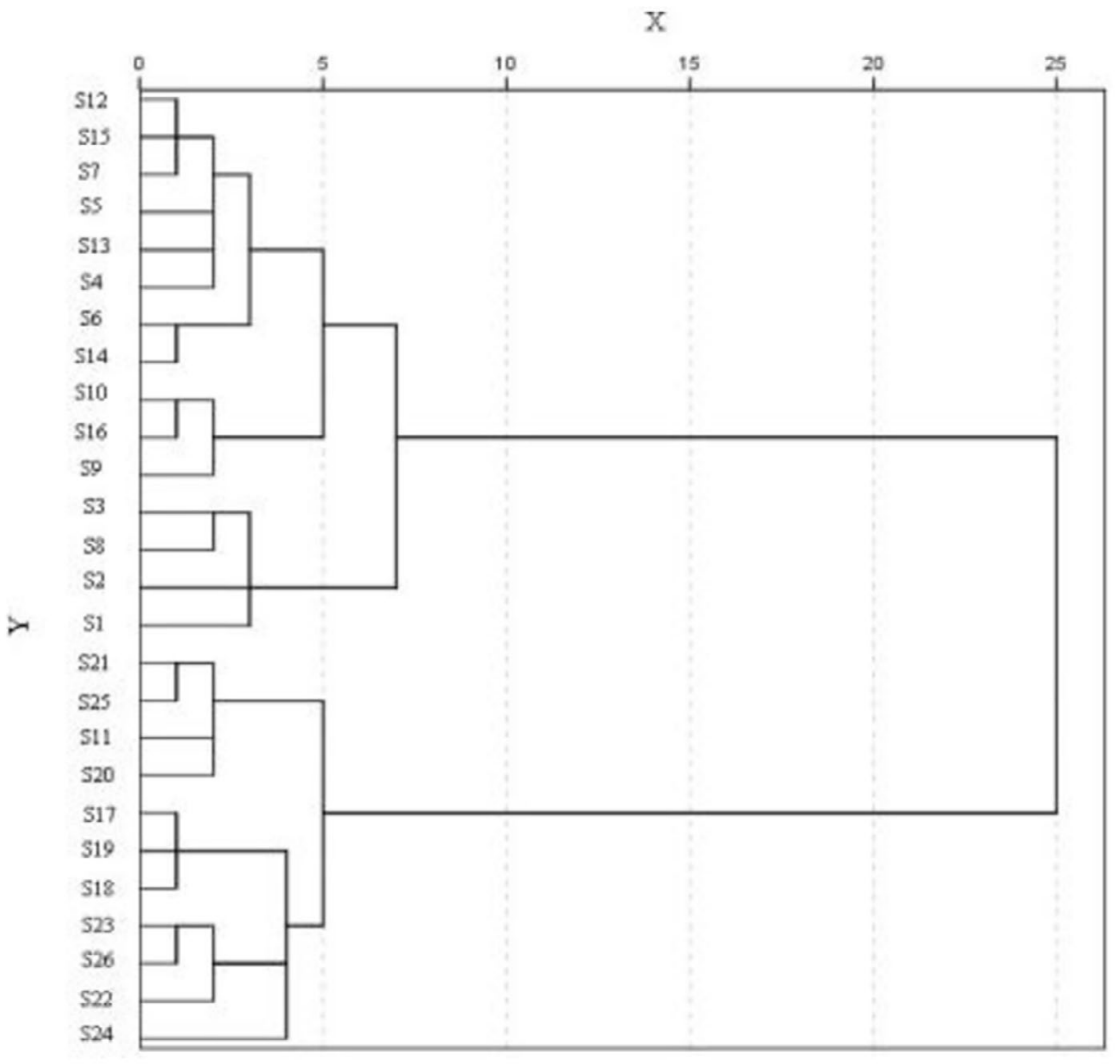
Cluster analysis of nutrients in leaves of different sea‐buckthorn varieties.

Cluster analysis was performed on the leaves of different varieties (strains) of sea buckthorn, and the tree diagram was obtained as shown in Figure 4. 26 sea‐buckthorn varieties (strains) can be divided into two categories. The first category includes 11 sea‐buckthorn varieties (strains), which are as follows: ‘S21’, ‘S25’, ‘S11’, ‘S20’, ‘S17’, ‘S19’, ‘S18’, ‘S23’, ‘S26’, ‘S22’, ‘S24’. The second category includes 15 sea‐buckthorn varieties (strains), which are as follows: ‘S12’, ‘S15’, ‘S7’, ‘S5’, ‘S13’, ‘S4’, ‘S6’, ‘S14’, ‘S10’, ‘S16’, ‘S9’, ‘S3’, ‘S8’, ‘S2’, ‘S1’. Among them, the Mg content in the leaves of the first type of sea‐buckthorn variety (strain) was higher than 2 mg/g, and the Mg content in the leaves of the second type of sea‐buckthorn variety (strain) was lower than 2 mg/g.

## 4 Discussion and Conclusion

From the study of flavonol composition, main nutrients, mineral elements, and secondary metabolites of 26 sea‐buckthorn germplasm leaves, there were three flavonols in 26 germplasm, and the relative content of flavonols in leaves of different germplasm was different. The relative content of flavonols was the lowest among the three flavonols, indicating that it was the first limiting flavonol in sea‐buckthorn leaves. The contents of isorhamnetin flavonols and quercetin flavonols were relatively high, which was consistent with the study results of Asofiei et al. (2019) on leaves of different varieties (strians) of sea‐buckthon and Raudone et al. (2021) on leaves of different varieties (strains) of sea‐buckthorn. The types of flavonol substances detected by this research institute differ to a certain extent from those identified by Dong et al. (2023). The experimental materials for this study were collected mainly in July from the Black Dragon River region at approximately 200 m above sea level. Additionally, Heilongjiang Province belongs to a temperate monsoon climate, with northwest areas having a temperate continental climate and southwest areas having a subtropical monsoon climate. This suggests that the types of flavonols in seabuckthorn leaves are not only related to variety characteristics but also closely associated with climatic conditions, geographical conditions, collection time, and other factors. Sea buckthorn leaves are widely utilized as food materials (sea buckthorn tea, etc.), and their flavonoid content determines the quality of the developed products.

In terms of main nutrients, there were obvious differences in the contents of crude protein, crude fiber, crude fat, and polysaccharide in the leaves of different sea‐buckthorn varieties (strains), while the differences in the contents of caffeine and VC were not obvious. The crude fiber content was higher than 9%, crude fat content was higher than 3%, polysaccharide content was higher than 4%, and crude protein content was higher than 15% in the leaves of most sea‐buckthorn varieties (strains). The crude protein content in the leaves of 26 sea‐buckthorn germplasm obtained by the test was significantly higher than that in alfalfa (Mortensen et al. 2018). Indicating that sea‐buckthorn leaves may be a high‐quality feed (Singh et al. 2020), added 3% sea‐buckthorn leaves to the basic diet of broilers. After systematically analyzing the growth performance, slaughter performance and blood biochemical indexes of broilers, they pointed out that the daily gain of broilers could be improved and the abdominal fat percentage significantly decreased in all the added groups, but the slaughtering performance, and biochemical indexes of the body had no significant effect. In the cross of *Hippophae rhamnoides subsp. sinensis Rousi × Hippophae rhamnoides subsp. mongolica* the Zn content of S10, S9, and S14 was higher than that of other varieties (lines), and the Mn content of S9, S10 was significantly higher than that of the other varieties (lines), which had superior performance in terms of nutrient content. Research has shown that zinc and manganese have a significant effect on plant growth, and when absorbed by the human body in the form of tea, they have a certain effect on human metabolism and disease prevention (López‐Rayo, Valverde, and Lucena 2023).

In terms of secondary metabolites, there were significant differences in the contents of total flavonoids and total polyphenols in the leaves of different sea‐buckthorn varieties (strains), and the contents of total flavonoids and total polyphenols in the leaves of most sea buckthorn varieties (strains) were higher than 2%. The contents of total polyphenols and flavonoids in sea buckthorn leaves are comparable to those of some high‐quality famous teas (Lv et al. 2021). This shows that the leaves of sea‐buckthorn in cold areas are important tea resources and can be roasted to produce tea with better quality. The main reasons for the better leaf quality of sea‐buckthorn in cold areas are: (1) species and variety characteristics of sea buckthorn, and (2) Unique climatic conditions.

In terms of mineral elements, the contents of Na, K, Ca, Mg, Fe, Mn, Cu, and Zn in the leaves of different sea‐buckthorn varieties (strains) are significantly different. The content of Na in the leaves of most sea‐buckthorn varieties (strains) is higher than 30 mg/100 g, the content of K is higher than 13 mg/100 g, and the content of Ca is higher than 117 mg/100 g. Mg content is higher than 120 mg/100 g, Fe content is higher than 17 mg/100 g, Mn content is higher than 10 mg/100 g, Cu content is higher than 1 mg/100 g, Zn content is higher than 7 mg/100 g. It was found that the contents of Fe, Ca, and Mn in the leaves of 26 sea‐buckthorn varieties (strains) were significantly higher than those of artificially cultivated sea‐buckthorn leaves (He et al. 2023). And the content of Zn was 6–27 times that of the leaves in Romania (Kim et al. 2021). This may be related to planting environment, cultivation technique, sampling site, leaf maturity, and harvest season.

As we all know, the content of various components in plants is closely related to varieties and ecological factors (Ilhan et al. 2021) and is also affected by cultivation techniques (Wang et al. 2022). In this study, only 26 varieties (strains) of sea‐buckthorn cultivated in cold areas were analyzed. Therefore, it cannot be pointed out that sea‐buckthorn leaves are good drinks or feeds overnight. The nutrient content in leaves of different varieties and regions should be comprehensively analyzed, and the feasibility of processing sea‐buckthorn leaves into drinks or feeds should be scientifically evaluated. To some extent, the leaves and fruits of sea buckthorn have broad application prospects, but the raw material characteristics are different, so the quality of processed products will be different. In the evaluation of sea‐buckthorn resources, the yield, growth, and development characteristics should also be considered on the basis of the analysis of nutritional indexes, so as to realize the scientific evaluation of sea buckthorn resources. At the same time, it is also necessary to consider the production mode of sea buckthorn products, produce different sea‐buckthorn products for different groups of people and different consumer classes, and strive to diversify sea buckthorn products and maximize economic benefits (Gutzeit, Winterhalter, and Jerz 2008).

## Author Contributions

**Yue Yuan:** formal analysis (equal), resources (equal), software (equal), supervision (equal), writing – original draft (equal), writing – review and editing (equal). **Wentao Yao:** conceptualization (equal), data curation (equal), investigation (equal), resources (equal), software (equal), validation (equal), writing – original draft (equal). **Ke Tang, Yuqi Wu and Rui Wang:** investigation and software. **Zeyuan Yu, Junwei Huo and Xingguo Li:** original draft.

## Conflicts of Interest

The authors declare no conflicts of interest.

## Data Availability

Even though adequate data have been given in the form of tables and figures, however, all authors declare that if more data are required, then the data will be provided on a request basis.
